# UCH-L1 down-regulates HER2 expression and increases lapatinib sensitivity by interacting with HSP90 in HER2-positive breast cancer

**DOI:** 10.1016/j.gendis.2025.101952

**Published:** 2025-11-24

**Authors:** Min Zheng, Xisha Chen, Wenqiang Gan, Lei Hou, Zhongmei Zhou, Ceshi Chen, Zhenzhen Liu, Yan Cheng

**Affiliations:** a Yunnan Key Laboratory of Animal Models and Human Disease Mechanisms, Kunming Institute of Zoology, Chinese Academy of Sciences, Kunming, Yunnan 650201, China; b Kunming College of Life Science, University of Chinese Academy of Sciences, Kunming, Yunnan 650204, China; c The Third Affiliated Hospital, Kunming Medical University, Kunming, Yunnan 650118, China; d Academy of Biomedical Engineering, Kunming Medical University, Kunming, Yunnan 650500, China; e Department of Breast Disease, Henan Breast Cancer Center, The Affiliated Cancer Hospital of Zhengzhou University & Henan Cancer Hospital, Zhengzhou, Henan 450008, China; f Department of Pharmacy, The Second Xiangya Hospital, Central South University, Changsha, Hunan 410011, China; g Hunan Provincial Engineering Research Centre of Translational Medicine and Innovative Drug, Changsha, Hunan 410011, China; h Cancer Research Institute, The First Affiliated Hospital, Hengyang Medical School, University of South China, Hengyang, Hunan 421001, China

HER2-positive breast cancer exhibits high aggressiveness, a propensity for recurrence, and a poor prognosis.^1^ Approximately 16%–22% of early stage patients still experience recurrence, and 22%–25% of patients with metastatic HER2^+^ breast cancer exhibit primary or acquired resistance. Therefore, it is necessary to investigate the molecular mechanisms that regulate HER2 status in order to develop new therapeutic strategies. Ubiquitin carboxyl-terminal hydrolase-L1 (UCH-L1), also known as protein gene product 9.5 (PGP9.5), is a member of the ubiquitin C-terminal hydrolase (UCH) family.^2^ UCH-L1 can play either a pro- or anti-tumorigenic role in different types of cancer because UCH-L1 acts by regulating different substrates. The overexpression of UCH-L1 has been observed to induce G0/G1 cell cycle arrest and apoptosis by stabilizing p53. In another independent study, UCH-L1 was observed to interact with AKT in MCF-7 cells, resulting in the up-regulation of phosphorylated AKT and an increase in the invasive capacity of MCF-7 cells. Therefore, it is crucial to investigate the mode of action of UCH-L1 in different cancers.^3^

In breast cancer, the role of UCH-L1 has been reported in both ER^+^ and triple negative breast cancer (TNBC),^4^^,^^5^ but its role in HER2^+^ breast cancer has not been reported. In the GEPIA2 database, UCH-L1 expression was lower in HER2^+^ breast cancer tissue than that in normal breast tissue (Fig. S1A). An analysis of the CCLE database revealed that UCH-L1 is expressed at low levels in both ER^+^ and HER2^+^ breast cancer cells and at high levels in TNBC (Fig. S1B). Concurrently, our results showed that the expression of UCH-L1 was barely detectable in HER2-overexpressing breast cancer cells (Fig. 1A). We analyzed 133 breast cancer samples and found that UCH-L1 was lowly expressed in HER2^+^ samples and highly expressed in HER2-samples, with a significant negative correlation (Fig. 1B). Following treatment of SK-BR-3 and MDA-MB-453 cells with the DNA methylation inhibitor decitabine, both UCH-L1 protein expression levels and transcriptional expression levels were up-regulated, indicating that the low expression of UCH-L1 in HER2^+^ breast cancer is caused by methylation (Fig. S1C and S1D). The Kaplan–Meier plotter database showed that patients with elevated UCH-L1 expression had prolonged relapse-free survival (RFS) (Fig. 1C). We then orthotopically injected nude mice with the HER2^+^ breast cancer cells, MDA-MB-453, and showed that UCH-L1 overexpression significantly inhibited tumor growth *in vivo* (Fig. S1E).

**Figure 1:**
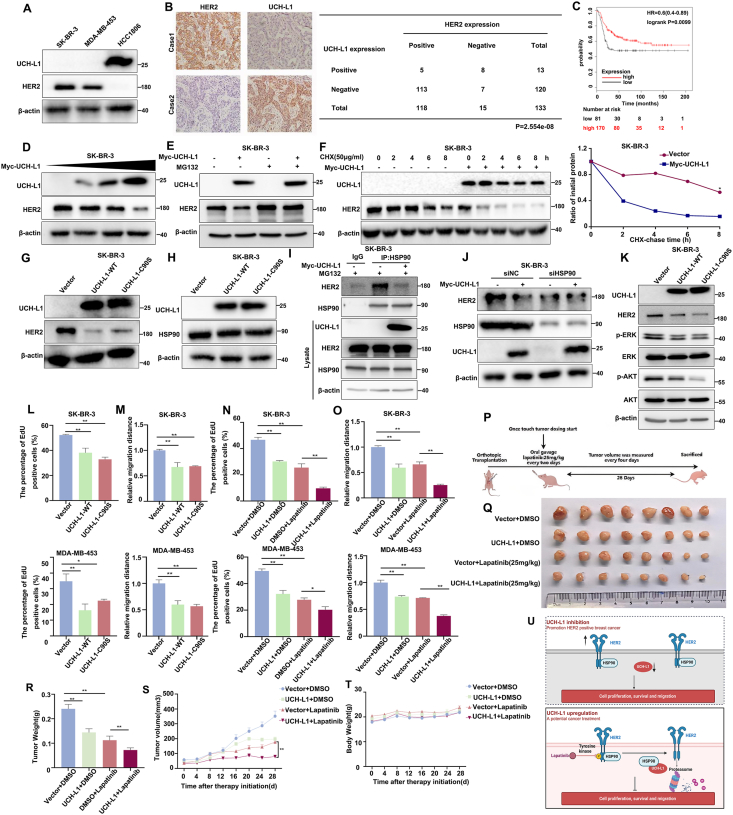
UCH-L1 down-regulates HER2 expression and increases lapatinib sensitivity by interacting with HSP90 in HER2-positive breast cancer. **(A)** UCH-L1 and HER2 protein expression in three breast cancer cells, as detected by Western blot. **(B)** IHC detection of UCH-L1 and HER2 protein expression in breast cancer tissues. The expression of UCH-L1 and HER2 protein was negatively correlated in 133 clinical breast cancer patients. **(C)** A high level of UCH-L1 mRNA expression is associated with good RFS in HER2^+^ breast cancer patients according to the data from the Kaplan–Meier Plotter database. **(D)** UCH-L1 decreased HER2 protein levels in SK-BR-3 cells in a dose-dependent manner. SK-BR-3 cells were transfected with increasing amounts of Myc-UCH-L1 plasmid (0, 0.5, 1.5, or 3 μg). HER2 protein expression was determined 48 h after transfection by Western blot. **(E)** UCH-L1 promoted HER2 protein degradation through the proteasome. SK-BR-3 cells were transfected with the Myc-UCH-L1 plasmid, followed by treatment with 20 μM MG132 for 4 h. The expression of UCH-L1 and HER2 was measured by Western blot. **(F)** UCH-L1 decreased the HER2 protein half-life. SKBR3 cells were transfected with Myc-UCH-L1. After treatment with 50 μg/mL cycloheximide (CHX) for the indicated times, the cells were harvested for Western blot. The band intensities were quantified using ImageJ software. **(G)** UCH-L1 down-regulated the HER2 protein level independent of its deubiquitinase activity. UCH-L1 WT and C90S mutant were overexpressed in SK-BR-3 cells. **(H)** UCH-L1 did not affect the HSP90 protein level. UCH-L1 WT and the C90S mutant were overexpressed in SK-BR-3 cells. **(I)** UCH-L1 decreased the interaction between HSP90 and HER2. UCH-L1 was overexpressed in SK-BR-3 cells, and MG132 was added at 20 μM for 4 h. Endogenous HSP90 protein was immunoprecipitated with an anti-HSP90 antibody. **(J)** UCH-L1 did not decrease the HER2 protein expression when HSP90 was depleted in SK-BR-3 cells. HSP90 siRNA was transfected to silence HSP90. **(K)** Overexpression of UCH-L1 WT and the C90S mutant in SK-BR-3 cells inhibited the HER2 signaling. **(L)** Overexpression of UCH-L1 WT and the C90S mutant in SK-BR-3 and MDA-MB-453 cells significantly decreased DNA synthesis, as determined by EdU assays. ∗*P* < 0.05, ∗∗*P* < 0.01. **(M)** Overexpression of UCH-L1 WT and the C90S mutant in SK-BR-3 and MDA-MB-453 cells significantly decreased cell migration. ∗*P* < 0.05, ∗∗*P* < 0.01. **(N)** UCH-L1 overexpression increased lapatinib sensitivity in SK-BR-3 and MDA-MB-453 cells. The cells were treated with lapatinib for 24 h, and DNA synthesis was determined via EdU assay. ∗*P* < 0.05, ∗∗*P* < 0.01. **(O)** SK-BR-3 and MDA-MB-453 cells were transfected with control and UCH-L1. Cell migration was determined by a cell migration assay. Representative images are shown. Scale bar, 200 μM ∗*P* < 0.05, ∗∗*P* < 0.01. (**P**) Working model of engraftment and treatment of the orthotopic model of MDA-MB-453. **(Q**–**T)** Overview of tumors, weights of tumor bulk, tumor volume, and body weight. ∗*P* < 0.05, ∗∗*P* < 0.01. **(U)** Mechanistic diagram of the regulation of HER2 by UCH-L1 to increase sensitivity to lapatinib.

Given that UCH-L1 is negatively correlated with HER2 expression in breast cancer, we asked whether UCH-L1 inhibits HER2 expression. As shown in Figure S2A, the overexpression of UCH-L1 significantly down-regulated HER2 expression in SK-BR-3 and MDA-MB-453 cells, and ectopically expressed UCH-L1 inhibited HER2 expression in a dose-dependent manner (Fig. 1D). To further validate whether endogenous UCH-L1 also suppresses HER2 expression, we knocked down UCH-L1 in HEK293T and three TNBC cells and found that the HER2 protein levels were up-regulated (Fig. S2B). However, UCH-L1 overexpression did not affect the HER2 mRNA levels (Fig. S2C). Based on these results, we suspected that UCH-L1 destabilizes the HER2 protein. Indeed, the proteasome inhibitor MG132 was able to rescue the down-regulation of HER2 protein expression induced by UCH-L1 overexpression (Fig. 1E; Fig. S2D). Furthermore, UCH-L1 overexpression decreased the half-life of the HER2 protein in a cycloheximide (CHX) assay (Fig. 1F). Consistently, the overexpression of UCH-L1 increased the HER2 ubiquitination levels (Fig. S2E). Immunoprecipitation experiments demonstrated no interaction between UCH-L1 and HER2, and that UCH-L1 increases the HER2 ubiquitination levels. Therefore, we hypothesize that UCH-L1 inhibits HER2 protein stability independently of its ubiquitin hydrolase activity. We overexpressed a UCH-L1 hydrolase-inactive mutant, UCH-L1 C90S, in SK-BR-3 and MDA-MB-453 cells. These results indicate that UCH-L1 functions independently on its deubiquitinating enzyme activity (Fig. 1G; Fig. S3A). Subsequent immunoprecipitation experiments showed no interaction between the UCH-L1 and HER2 proteins (Fig. S3B). These results suggest that UCH-L1 indirectly regulates HER2 protein expression.

We performed an IP-MS experiment and identified HSP90 as a UCH-L1-interacting protein (Fig. S3C). In the GEPIA2 database, HSP90 expression was higher in HER2^+^ breast cancer than in normal breast tissue (Fig. S3D). We then overexpressed UCH-L1 and UCH-L1 C90S in SK-BR-3 and MDA-MB-453 cells and found that neither affected the HSP90 protein expression levels (Fig. 1H; Fig. S3E). HSP90 ubiquitination was also not affected by UCH-L1 overexpression in HEK293T cells (Fig. S3F). HSP90 is a vital chaperone protein with a conserved binding site that interacts directly with HER2, maintaining its correct folding, maturation and conformation. This binding protects HER2 from proteasomal degradation, thereby ensuring its sustained signaling activity. UCH-L1 binds to HSP90 without affecting its expression. Therefore, we speculated that UCH-L1 and HER2 may compete for HSP90 so that UCH-L1 overexpression leads to HER2 proteasomal degradation due to the loss of HSP90 protection. To test this, we performed an immunoprecipitation experiment with a HSP90 antibody. As expected, UCH-L1 overexpression decreased the interaction between HER2 and HSP90 (Fig. 1I). Next, we simultaneously silenced HSP90 expression and overexpressed UCH-L1 in SK-BR-3 and MDA-MB-453 cells and found that UCH-L1 overexpression could no longer reduce HER2 protein levels in the absence of HSP90 (Fig. 1J; Fig. S3G). Previous studies in Parkinson’s disease have reported that the UCH-L1 I93M mutant exhibits enhanced interaction with HSP90. We overexpressed both UCH-L1 and UCH-L1 I93M in SK-BR-3 cells and showed that UCH-L1 I93M reduced HER2 protein levels more effectively compared to WT UCH-L1 (Fig. S4A). Consistently, the UCH-L1 I93M mutant reduced the interaction between HER2 and HSP90 more strongly than WT UCH-L1 did (Fig. S4B), and the mutant increased more ubiquitination of the HER2 protein than WT UCH-L1 did (Fig. S4C). In addition, we also overexpressed HER2 in SK-BR-3 cells and found that the inhibition of cell proliferation by UCH-L1 overexpression was rescued by HER2 overexpression. This suggests that UCH-L1 acts through HER2 (Fig. S4D). In HEK293T cells overexpressing UCH-L1, we examined changes in other HSP90 client proteins, such as CyclinD, CDK4, CDK6 and AKT, and the results showed that UCH-L1 is specific for HER2 degradation (Fig. S4E). To further investigate the interaction details among UCH-L1, HER2, and HSP90, we constructed three Glutathione S-Transferase (GST) fused truncated forms of HSP90 (Fig. S5A) and three GST fused truncated forms of HER2. Using GST pulldown assays, we found that HSP90 primarily binds to the intracellular kinase domain of HER2 (Fig. S5B). We then used the GST fused truncated forms of HSP90 to identify the domain of HER2 and UCH-L1 that bind to HSP90. We found that HER2 mainly binds to the N-terminus of HSP90 (Fig. S5C) and UCH-L1 mainly binds to the C-terminus of HSP90 (Fig. S5D). We further confirmed that the 30–60 aa region of UCH-L1 binds to HSP90 (Fig. S5E-S5G).

We examined the HER2 downstream signaling, including p-ERK and p-AKT protein levels. As expected, UCH-L1 and its mutant not only down-regulated the HER2 protein levels but also inhibited p-ERK and p-AKT levels (Fig. 1K; Fig. S6A). In addition, the overexpression of both UCH-L1 and its mutant UCH-L1 C90S significantly reduced the proliferation and DNA synthesis of SK-BR-3 and MDA-MB-453 cells (Fig. 1L; Fig. S6B and S6C). Consistently, UCH-L1 and UCH-L1 C90S significantly decreased the survival and migration of the SK-BR-3 and MDA-MB-453 cells (Fig. 1M; Fig. S6D and S6E). We wondered whether UCH-L1 could increase the sensitivity of lapatinib by inhibiting HER2 expression. We found that the overexpression of UCH-L1 alone or treatment with lapatinib in SK-BR-3 and MDA-MB-453 significantly inhibited the level of cell proliferation and DNA synthesis, while in the lapatinib-treated and UCH-L1-overexpressed group, the cell proliferation ability and DNA synthesis were further inhibited (Fig. 1N; Fig. S7A and S7B). Consistently, the combination of UCH-L1 overexpression and lapatinib exhibited a more pronounced inhibition of cell survival and cell migration in these cell lines compared to the lapatinib-treated group alone and the UCH-L1 overexpression group (Fig. 1O; Fig. S7C and S7D). The above results suggest that UCH-L1 may increase the sensitivity of HER2^+^ breast cancer to lapatinib. We also overexpressed UCH-L1 in MDA-MB-453 cells and orthotopically injected these cells into the fat pads of nude mice. After the tumors were visible, we treated the nude mice with lapatinib (25 mg/kg, every two days by oral gavage) (Fig. 1P). Consistent with the *in vitro* results, UCH-L1 overexpression significantly increased the sensitivity to lapatinib (Fig. 1Q–S), and there were no significant changes in the body weights of the mice (Fig. 1T).

In this study, we discovered that UCH-L1 inhibited cell proliferation, survival, migration, and tumor growth by binding to HSP90 and promoting the ubiquitination-mediated degradation of HER2. UCH-L1 reduces HER2 expression independently of its hydrolase activity. Furthermore, UCH-L1 enhances the inhibitory effect of lapatinib on HER2^+^ breast cancer. UCH-L1 expression was associated with a better prognosis in HER2^+^ breast cancer patients (Fig. 1U). Our findings provide new therapeutic ideas for the treatment of HER2^+^ breast cancer.

## CRediT authorship contribution statement

**Min Zheng:** Writing – original draft, Resources, Project administration, Methodology, Investigation, Formal analysis, Data curation. **Xisha Chen:** Supervision, Resources, Methodology. **Wenqiang Gan:** Methodology. **Lei Hou:** Methodology. **Zhongmei Zhou:** Resources. **Ceshi Chen:** Writing – review & editing, Supervision, Funding acquisition. **Zhenzhen Liu:** Writing – review & editing, Funding acquisition. **Yan Cheng:** Writing – review & editing, Supervision.

## Ethics declaration

This animal experiment was approved by the Animal Ethics Committee of Kunming Institute of Zoology, CAS (IACUC-RE-2024-12-003). IHC analysis of the specimen was approved by the Medical Ethics Committee of Henan Cancer Hospital, Zhengzhou University, Henan, China (2024-KY-0085). We have obtained informed consent from the patients.

## Funding

This work was supported by the 10.13039/501100012166National Key R&D Program of China (No. 2023ZD0502200, 2020YFA0112300), the 10.13039/501100001809National Science Foundation of China (No. U2102203, 82430084, 82372712), the Biomedical Projects of Yunnan Key Science and Technology Program (China) (No. 202302AA310046), Yunnan Basic Research Projects (No. 202201BC070002), and Yunnan Revitalization Talent Support Program (Yunling Scholar Project), Yunnan (Kunming) Academician Expert Workstation (No. YSZJGZZ-2020025), the Innovative Research Team of Yunnan Province, China (No. 202405AS350016), and the 10.13039/501100017700Henan Province Science and Technology Research Project (China) (No. 242102311027).

## Conflict of interests

Ceshi Chen is a member of *Genes & Diseases* Editorial Board. To minimize bias, he was excluded from all editorial decision-making related to the acceptance of this article for publication. The remaining authors declare no conflict of interests.
