# Supplementary material for: UCH-L1 down-regulates HER2 expression and increases lapatinib sensitivity by interacting with HSP90 in HER2-positive breast cancer
Source: Genes Dis. 2025 Nov 24;13(4):101952. doi: 10.1016/j.gendis.2025.101952 (PMC13011018; doi:10.1016/j.gendis.2025.101952)
Supplement: Multimedia component 1 [file mmc1.docx]

**Supplementary Materials**

**Supplementary Figure 1**

**Supplementary Figure 2**

**Supplementary Figure 3**

**Supplementary Figure 4**

**Supplementary Figure 5**

**Supplementary Figure 6**

**Supplementary Figure 7**

**Supplementary Materials and Methods**

**
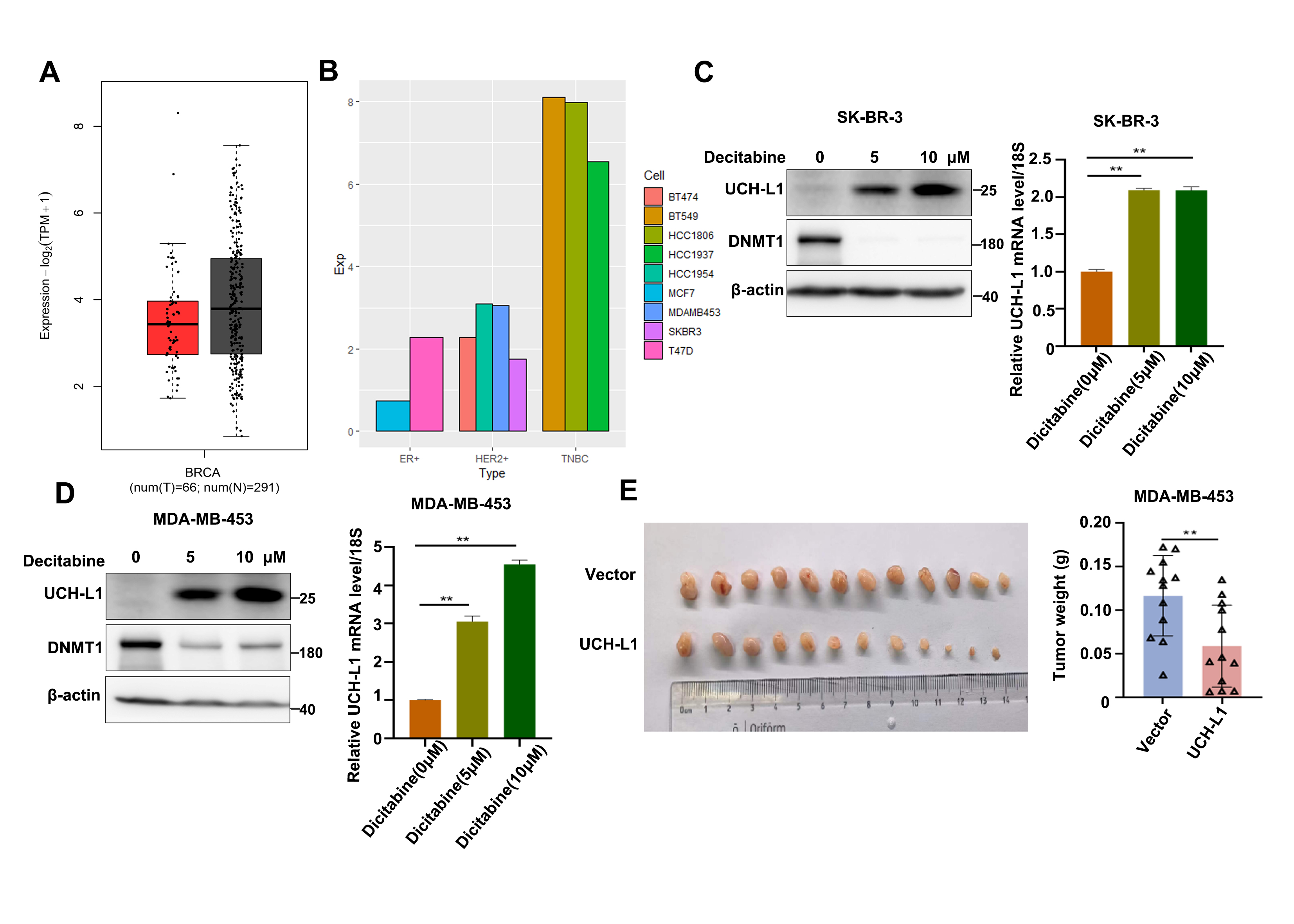
**

**Fig. S1 UCH-L1 is negatively correlated with HER2 expression and inhibits tumor progression in HER2+ breast cancer.**

(A) The GEPIA2 database was used to evaluate UCH-L1 expression in HER2+ breast cancer. (B) The CCLE database was used to analyze the expression of UCH-L1 in cell lines of different breast cancer subtypes. (C) UCH-L1 protein and mRNA expression were detected in SK-BR-3 cells treated with decitabine. (D) UCH-L1 protein and mRNA expression were detected in MDA-MB-453 cells treated with decitabine. (E) UCH-L1 suppressed MDA-MB-453 xenograft tumor growth in female nude mice. The cells stably overexpressing UCH-L1 were transplanted into the fat pads of 5-week-old mice. Weights of tumor bulk. ***P*<0.01.


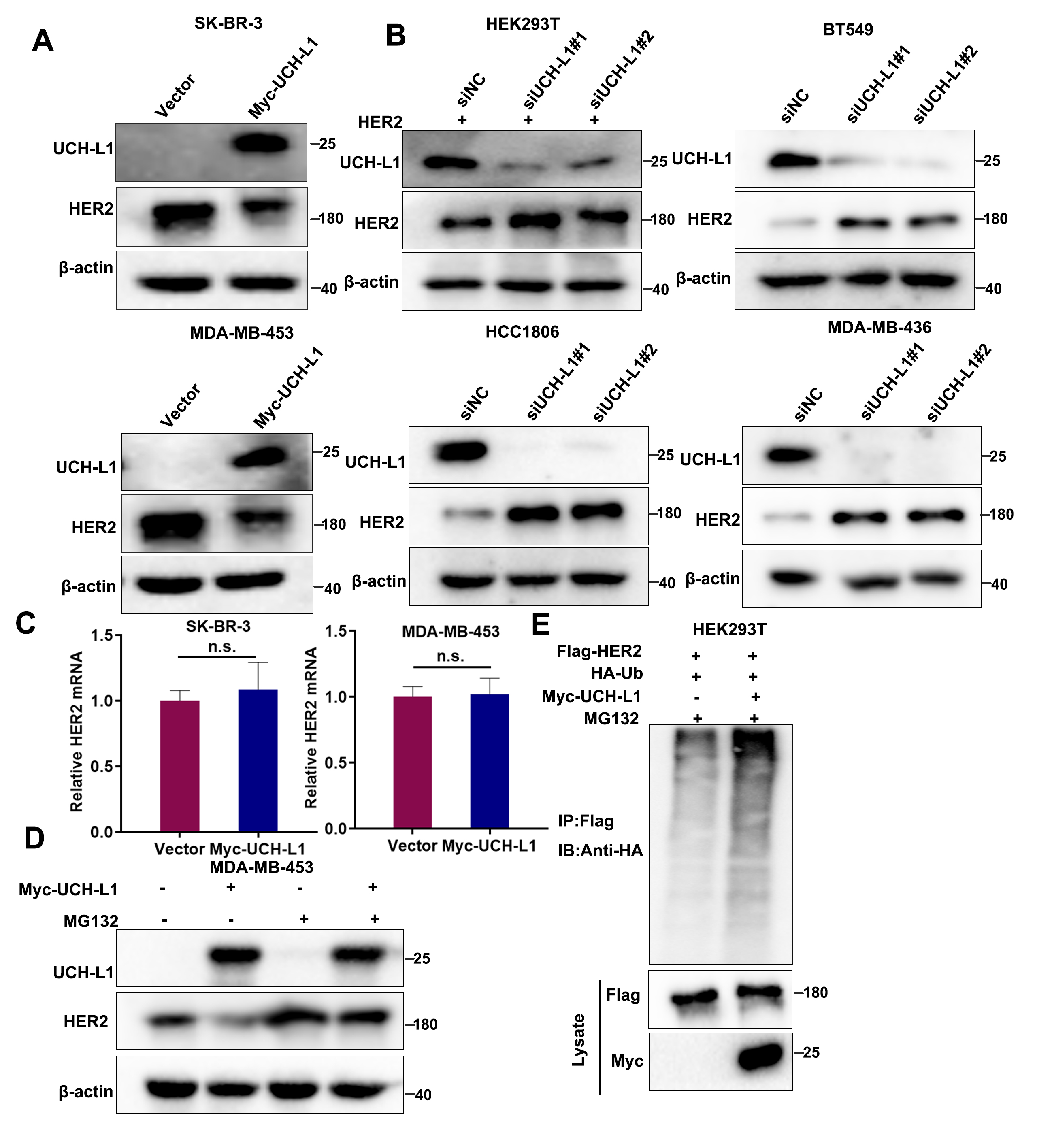


**Fig. S2 UCH-L1 promotes HER2 protein ubiquitination and proteasomal degradation.**

(A) UCH-L1 decreased HER2 protein levels in SK-BR-3 and MDA-MB-453 cells. The cells were transfected with Myc-UCH-L1 for 48 hours. (B) HER2 protein expression levels were examined in cells overexpressing UCH-L1 by knocking down UCH-L1. (C) HER2 mRNA expression levels were examined in SK-BR-3 and MDA-MB-453 cells overexpressing UCH-L1. (D) UCH-L1 promoted HER2 protein degradation through the proteasome. MDA-MB-453 cells were transfected with the Myc-UCH-L1 plasmid, followed by treatment with 20 µM MG132 for 4 hours. The expression of UCH-L1 and HER2 were measured by Western blot. (E) UCH-L1 increased HER2 protein ubiquitination. Flag-HER2, Myc-UCH-L1 and HA-Ub plasmids were co-expressed in HEK293T cells. Cells were treated with 20 µM MG132 for 4 hours and harvested for immunoprecipitation with anti-Flag antibody and ubiquitination detection.


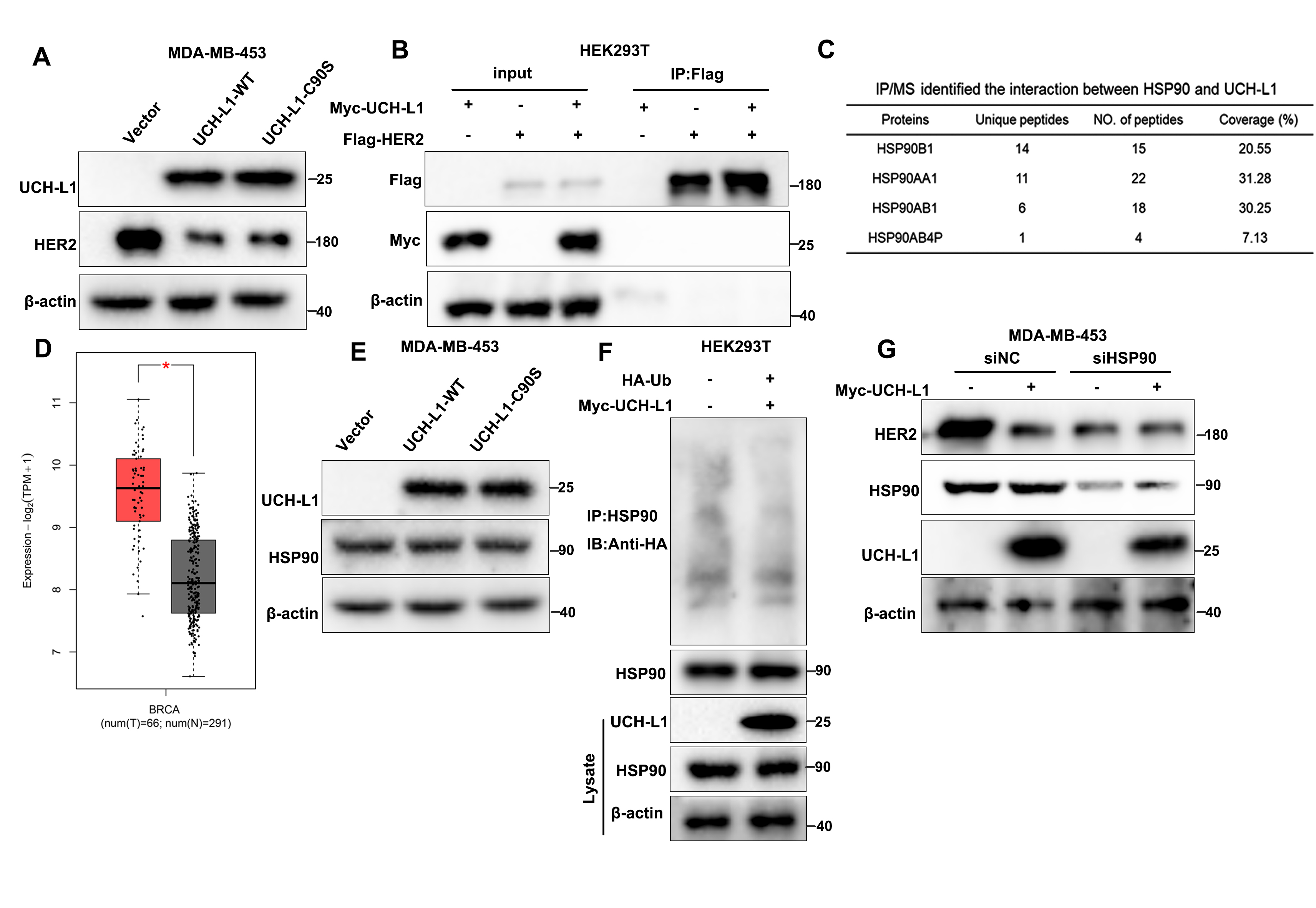


**Fig. S3 UCH-L1 interacts with HSP90 to downregulate the HER2 protein level independent of its hydrolase activity.**

(A) UCH-L1 downregulated the HER2 protein level independent of its deubiquitinase activity. UCH-L1 WT and C90S mutant were overexpressed in MDA-MB-453 cells. (B) Co-expression of Myc-UCH-L1 and Flag-HER2 plasmids in HEK293T cells. Cells were harvested for immunoprecipitation. (C) IP-MS results showed the interaction between UCH-L1 and HSP90. (D) The GEPIA2 database was used to evaluate HSP90 expression in HER2+ breast cancer. **P*<0.05. (E) UCH-L1 did not affect the HSP90 protein levels. UCH-L1 WT and C90S mutant were overexpressed in MDA-MB-453 cells. (F) Co-expression of Myc-UCH-L1 and HA-Ub plasmids in HEK293T cells. Cells were harvested, immunoprecipitated with anti-HSP90 antibody and assayed for ubiquitination. (G) UCH-L1 did not decrease the HER2 protein expression when HSP90 was depleted in MDA-MB-453 cells. HSP90 siRNA was transfected to silence HSP90.


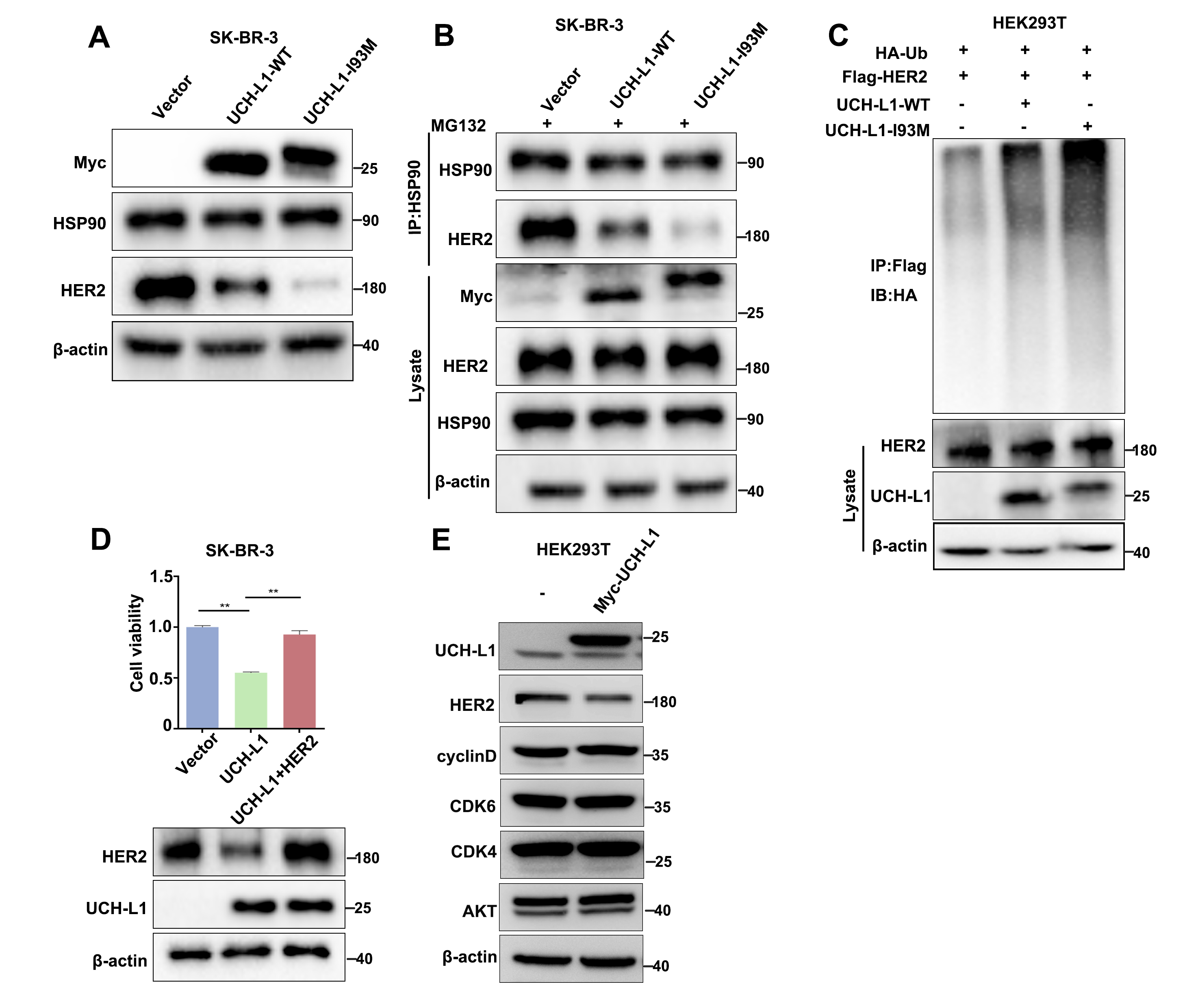


**Fig. S4** **UCH-L1 specifically downregulates HER2 expression by inhibiting the interaction between HSP90 and HER2.**

(A) Overexpression of UCH-L1 I93M mutant in SK-BR-3 cells decreased HER2 protein expression more than WT did. (B) UCH-L1 I93M mutant decreased HSP90-HER2 interaction more than WT did. SK-BR-3 cells overexpressing UCH-L1 WT and I93M mutant. MG132 was added at 20 µM for 4 hours prior to harvest. Endogenous HSP90 protein was immunoprecipitated with anti-HSP90 antibody. (C) UCH-L1 I93M mutant increased HER2 ubiquitination more than WT did. Flag-HER2, WT and I93M mutant UCH-L1 and HA-Ub plasmids were coexpressed in HEK293T cells. Cells were treated with 20 µM MG132 for 4 hours. Cells were harvested for immunoprecipitation with anti-Flag antibody and ubiquitination detection. (D) SK-BR-3 cells overexpressing UCH-L1 and HER2. Cell viability was measured by CCK-8 assay. **P*<0.05, ***P*<0.01. (E) Overexpression of UCH-L1 in HEK293T to detect HSP90 client proteins expression.


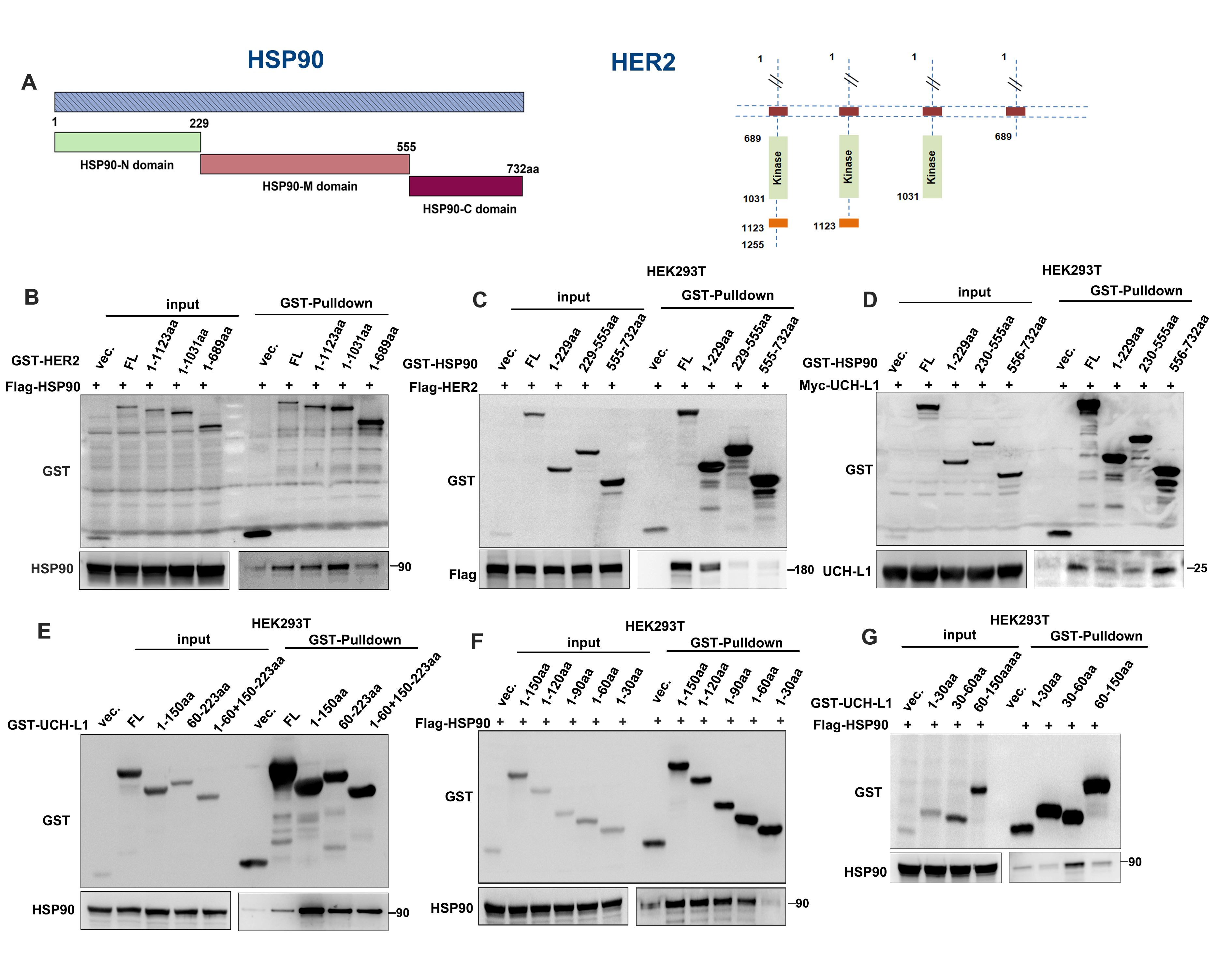


**Fig. S5 The interaction details among UCH-L1, HSP90 and HER2.**

(A) Diagrams of GST-fused HSP90 and HER2 fragments. (B) HSP90 mainly binds to the kinase domain of HER2. Flag-tagged full-length HSP90 was transfected into HEK293T cells with different GST-tagged full-length or truncated HER2. After 48 hours of transfection, cells were harvested for GST pulldown assays. (C) HER2 mainly binds to the N terminus of HSP90. Flag-tagged full-length HER2 was transfected into HEK293T cells with different GST-tagged full-length or truncated HSP90. After 48 hours of transfection, cells were harvested for GST pulldown assays. (D) UCH-L1 mainly binds to the C terminus of HSP90. Myc-tagged full-length UCH-L1 was transfected into HEK293T cells with different GST-tagged full-length or truncated HSP90. After 48 hours of transfection, cells were harvested for GST pulldown assays. (E-G) The 30-60 aa region of UCH-L1 binds to HSP90. HEK293T cells were transfected with different GST-tagged full-length or truncated UCH-L1, cells were harvested for GST pulldown assays.

**
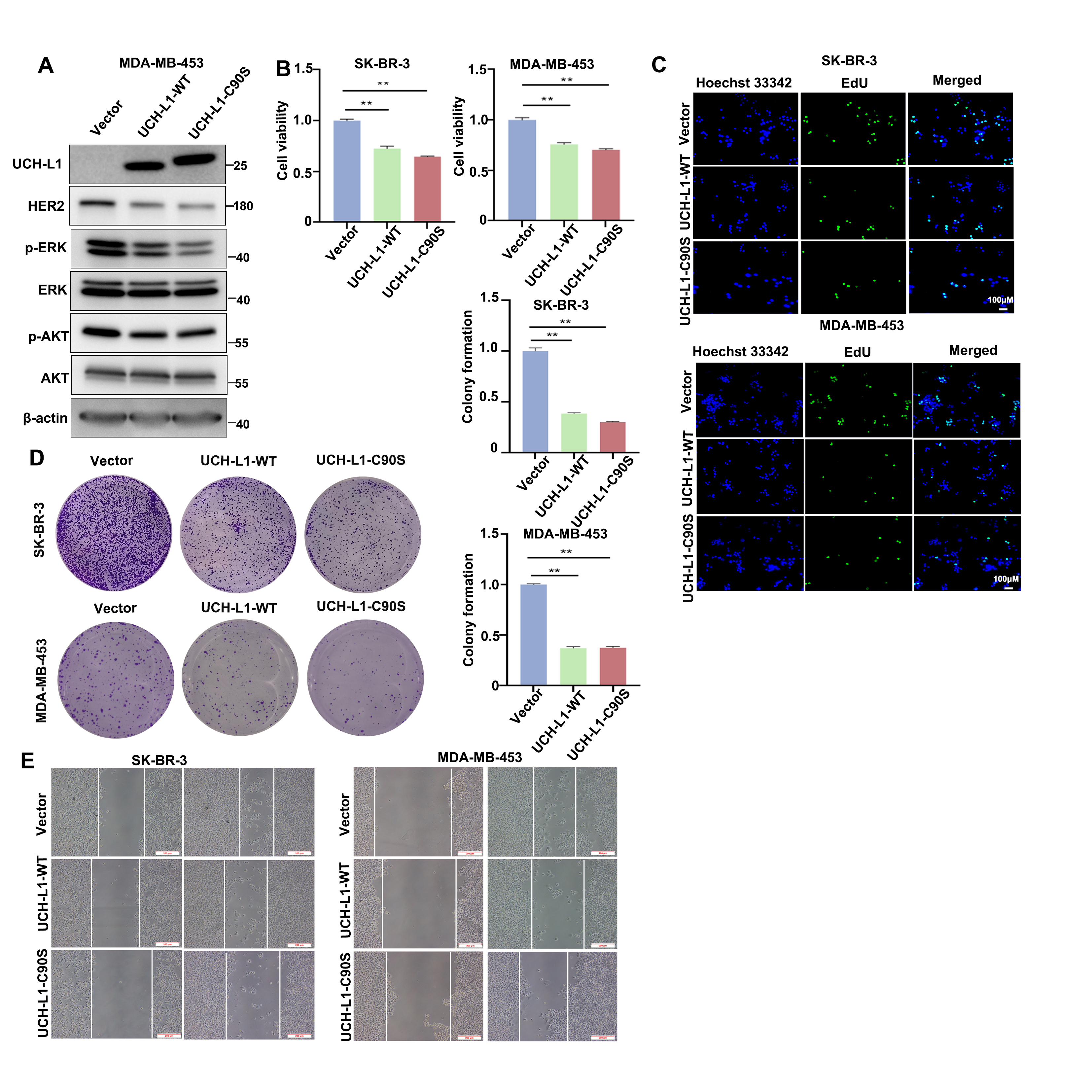
**

**Fig. S6 UCH-L1 inhibited proliferation and migration of HER2-positive breast cancer cells in a hydrolase activity-independent manner.**

(A) Overexpression of UCH-L1 WT and C90S mutant in MDA-MB-453 cells inhibited the HER2 signaling. (B) Overexpression of UCH-L1 WT and C90S mutant in SK-BR-3 and MDA-MB-453 cells significantly decreased cell viability, as measured using CCK-8 assay. **P*<0.05, ***P*<0.01.

(C) Overexpression of UCH-L1 WT and C90S mutant in SK-BR-3 and MDA-MB-453 cells significantly decreased DNA synthesis, as determined by EdU assays. Representative images are shown. **P*<0.05, ***P*<0.01. (D) Overexpression of UCH-L1 WT and C90S mutant in SK-BR-3 and MDA-MB-453 cells significantly decreased cell survival, as determined by colony formation assay. **P*<0.05, ***P*<0.01. (E) SK-BR-3 and MDA-MB-453 cells, overexpression of UCH-L1 wild-type and C90S mutant UCH-L1. Cell migration was determined by cell migration assay. Representative images are shown. Scale bar, 200 µM.

**
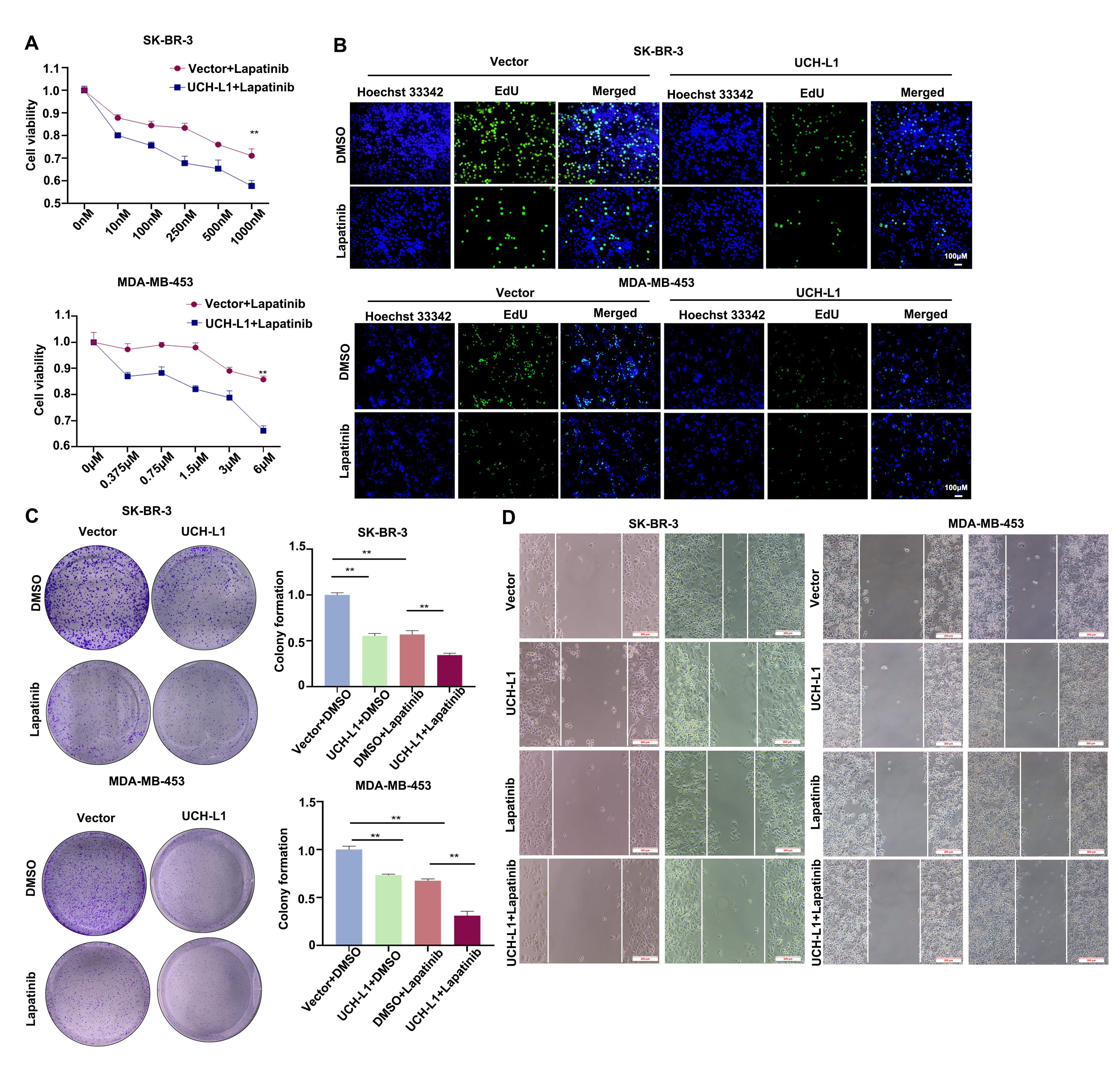
**

**Fig. S7 UCH-L1 promotes lapatinib sensitivity in HER2+ breast cancer cells.**

(A) UCH-L1 overexpression increased lapatinib sensitivity in SK-BR-3 and MDA-MB-453 cells. Cell viability was measured by CCK-8 assay. **P*<0.05, ***P*<0.01. (B) UCH-L1 overexpression increased lapatinib sensitivity in SK-BR-3 and MDA-MB-453 cells. The cells were treated with lapatinib for 24 hours, DNA synthesis was determined by EdU assay. Representative images are shown. **P*<0.05, ***P*<0.01. (C) UCH-L1 overexpression increased lapatinib sensitivity in SK-BR-3 and MDA-MB-453 cells. Cell survival was determined by colony formation assay. **P*<0.05, ***P*<0.01. (D) SK-BR-3 and MDA-MB-453 cells were transfected with control and UCH-L1. Cell migration was determined by cell migration assay. Representative images are shown. Scale bar, 200 µM. **P*<0.05, ***P*<0.01.

**Supplementary Materials and Methods**

**Cell Culture and Reagents**

The human breast cancer cell lines SK-BR-3 and MDA-MB-453 were obtained from the American Type Culture Collection (ATCC). The SK-BR-3 cell line was cultured in RPMI-1640 (Gibco, C11875500BT) supplemented with 10% fetal bovine serum (FBS) (ExCell Bio, FSD500). The MDA-MB-453 cell line was cultured in high glucose Dulbecco's modified Eagle's medium (DMEM) (Gibco, C11995500BT) supplemented with 10% FBS (ExCell Bio, FSD500). All cell lines were maintained in a 5% CO2 incubator at 37°C. We authenticated the cell lines by short tandem repeat (STR) assays before starting the experiments. MG132 was purchased from Sigma (USA, 474790). Cycloheximide (CHX) was purchased from Amresco (Solon, OH, USA). Lapatinib (MedChemExpress, Monmouth Junction, NJ, USA, HY-50898) is an inhibitor of the tyrosine kinase structural domain of HER2.

**Western Blot and Antibodies**

Cells were harvested, lysed in RIPA lysis buffer supplemented with 1× proteinase inhibitor cocktail (MedChemExpress, Monmouth Junction, NJ, USA, HY-K0010), and quantified using a BCA kit (Thermo Fisher). Briefly, ~40 µg of each protein sample was subjected to SDS-PAGE and transferred to polyvinylidene fluoride (PVDF) membranes (Merck Millipore, Germany, #IPFL00010). The membrane was blocked with 5% milk for one hour and then incubated with primary antibody overnight at 4°C. The membrane was incubated with horseradish peroxidase (HRP)-conjugated secondary antibodies (Invitrogen, #31460 & #31430) for 1 hour at room temperature. Chemiluminescence was detected using an ImageQuant LAS4000 biomolecular imager (GE, USA) with Western HRP substrate (US Everbright, S6009 L). The following primary antibodies were used: anti-UCH-L1 (cell signaling technology, 13179), anti-GST (Proteintech, 10000-0-AP), anti-β-actin (Proteintech, 60008-1-lg), anti-Myc (Proteintech, 16286-1-AP), anti-Flag (Proteintech, 66008-3-lg), anti-HA (cell signaling technology, 3724), anti-HER2 (cell signaling technology, 2165), anti-HSP90 (Proteintech, 60318-1-lg).

**Transfection and Production of Lentiviral Particles**

To construct stably overexpressing UCH-L1 cells, HEK293T cells were transfected with PCDH-UCH-L1 or empty, and the packaging plasmids. Lentiviruses were harvested after 48 hours and stored at -80°C. Lentiviruses were added to MDA-MB-453 cells along with 8 µg/mL polypyrene (Sigma, H9268), and after 24 hours, the medium was replaced with fresh medium supplemented with 1 µg/mL puromycin (InvivoGen) to select for stably infected cells.

**Real-time quantitative PCR (RT-qPCR)**

RNA samples were extracted with TRIzol reagent (Invitrogen, 15596018). Reverse transcription was performed using HiScript II Q RT SuperMix for qPCR (Vazyme, R223-01), and quantitative reverse transcriptase PCR was performed using Taq Pro Universal SYBR qPCR Master Mix (Vazyme, Q712-02). Primer sequences for UCH-L1 and 18S were as follows: UCH-L1 forward, 5′- GCCAATGTCGGGTAGATG -3′ and UCH-L1 reverse, 5′- CAA AGTCCCTCCCACAGA-3′; 18S forward, 5′- CTCAACACGGGAAACCTCAC-3′ and 18S reverse, 5′-CGCTCCACCAACTAAGAACG-3′; HER2 forward, 5′-AGCCGCGAGCACCCAAGT-3′ and HER2 reverse, 5′-TTGGTGGGCAGGTAGGTGAGTT-3′.

**Pulse-chase Assay**

For the HER2 half-life assay, UCH-L1 plasmid was transfected into SK-BR-3 cells when the cells reached approximately 60% confluence. Twenty-four hours later, the cells were treated with the protein synthesis inhibitor cycloheximide (Amresco, 50 µg/ml) for the indicated times before collection.

**Immunoprecipitation**

For immunoprecipitation, SK-BR-3 cells were transfected with Myc-UCH-L1 or empty vector using Lipo2000 (Invitrogen, 11668019). After 48 hours, the cells were harvested and lysed with lysis buffer (50 mM Tris, 150 mM NaCl, 1 mM EDTA, 1% NP-40, 10% glycerol; pH 7.5). A protease inhibitor cocktail was added to the cell lysate. SK-BR-3 cells were lysed with lysate followed by mouse normal IgG or HSP90 antibody and incubated overnight at 4°C. Antibody-coupled lysates were incubated with Protein A/G beads (MedChemExpress, Monmouth Junction, NJ, USA, HY-K0202) for 2 hours. The beads were thoroughly washed and eluted, and the supernatants were analyzed by Western blotting as described above.

**GST Pulldown**

The interaction between GST-UCH-L1 and Flag-HSP90 was demonstrated by co-transfecting HEK293T cells with the overexpression plasmids of GST-UCH-L1 and Flag-HSP90. The cells were lysed for 30 minutes on ice, and the lysate was collected and centrifuged at 13,000 rpm and 4 ℃ for 15 minutes, and the supernatant was mixed with glutathione sepharose 4B slurry beads in a vertical mixer at 4℃ 360 degrees for 2-4 hours. After incubation, wash the beads three times with lysate and centrifuge at 500 g for 1 minutes to remove the supernatant. 40 µL SDS buffer was added and the samples were subjected to Western blotting.

**Clonogenic Assays**

Cells were grown in 6-well tissue culture plates (500 cells per well) and incubated at 37°C for 15 days in a humidified environment with 5% CO2. At the end of the incubation period, the cells were fixed with 4% paraformaldehyde for 30 minutes, washed, and air dried. The cells were then stained with crystal violet for 20 minutes and washed. Finally, the cells were photographed and the colonies were counted.

**EdU Assays**

After treatment, cells were subjected to EdU treatment for 2 hours. The cells were then fixed with 4% paraformaldehyde for 30 minutes. 50 µL of 2 mg/mL glycine solution was added to each well and incubated for 5 minutes at room temperature, and the cells were washed twice with 100 µL of 3% BSA per well. The wash solution was removed and 100 µL of 0.5% Triton X-100 was added and incubated for 20 minutes at room temperature. Click-iT Reaction Mix (Uelandy, C6043M) was added and incubated for 30 minutes at room temperature. The cells were washed twice with 100 µL of 3% BSA per well, and then 100 µL of 1× Hoechst 33342 solution was added to each well, incubated for 15-30 minutes at room temperature in dark, the Hoechst 33342 solution was removed, and the cells were washed twice with 100 µL of PBS. The cells were washed twice with 100 µL PBS. Immediately after staining, the cells were photographed under a fluorescence microscope.

**Cellular Viability Assays**

Cells at 8×10^3^ cells per well were plated in 96-well tissue culture plates with different treatments and then incubated at 37°C in a humidified environment with 5% CO2 air for the indicated times. Cell viability was determined using the CCK-8 assay.

**Cell Migration Assays**

To evaluate the migration of SK-BR-3 and MDA-MB-453 cells after overexpression of UCH-L1 and after overexpression of UCH-L1 in combination with lapatinib, wound healing assays were performed. The cells were scratched 24 hours after seeding. Wound closure was then observed and recorded under a microscope. The width of the gap was then quantified in each image using Image J software.

**Xenograft Animal Studies**

MDA-MB-453 cells stably overexpressing UCH-L1 (6×10^6^/point) were suspended in 75 µL of Matrigel (Corning, BD Biocoat, #354234) and phosphate-buffered saline (PBS) at a 1:1 ratio and injected into the fat pads of 5-week-old BALB/C nude mice obtained from Hunan SJA Laboratory Animal Co. Ltd. (Changsha, Hunan, China). This animal experiment was approved by the Animal Ethics Committee of Kunming Institute of Zoology, CAS (IACUC-RE-2024-12-003). Tumor volume was calculated as follows: Tumor volume = π × (length × width^2^)/6. When the tumors reached the tenth day of growth, each group of mice was randomly and equally divided into two subgroups. The two subgroups were treated with drug control or 25 mg/kg lapatinib (once every two days) by gavage, respectively. At the end of the experiment, all mice were sacrificed and tumors were harvested for analysis.

**Statistical Analysis**

Data are expressed as mean ± SD. Statistical analyses were performed using Student's t-test, unless otherwise noted. GraphPad Prism 8 (GraphPad Software Inc., La Jolla, CA, USA) was used for all statistical analyses. P values less than 0.05 were considered significant; **, P < 0.01; *, P < 0.05; ns, not significant, t-test.
